# Mowat-Wilson syndrome: Case report

**DOI:** 10.1097/MD.0000000000039082

**Published:** 2024-07-19

**Authors:** Bogumiła Wójcik-Niklewska, Erita Filipek

**Affiliations:** a Department of Pediatric Ophtalmology, Faculty of Medical Sciences in Katowice, Medical University of Silesia in Katowice, Katowice, Poland; b Kornel Gibiński University Clinical Centre, Katowice, Poland.

**Keywords:** children, Mowat-Wilson, ocular abnormalities, ZEB2 gene

## Abstract

**Background:**

Mowat-Wilson syndrome (MWS) is a rare genetic condition resulting in multiple congenital anomalies, including facial dysmorphism, structural anomalies of the internal organs, functional disorders, and, although less commonly, ocular abnormalities. To present a child with MWS and eye abnormalities.

**Methods:**

A 3-year-old boy was born at 37 weeks of pregnancy with dysmorphic features, neurodevelopmental disorders, genetically confirmed MWS, nystagmus, strabismus, and suspicion of congenital glaucoma. Ophthalmic examination was carried out under general anesthesia; eyeball ultrasound and electrophysiological examination (flash visual evoked potentials) were also performed.

**Results:**

The examinations revealed nystagmus, a normal response of pupils to light in both eyes, and normal intraocular pressure, that is, 17 and 18 mm Hg in the right and left eye, respectively. Corneal thickness was 606 µm in the right eye and 588 µm in the left eye. Gonioscopy revealed displacement of Schwalbe line anterior to the limbus of the cornea (posterior embryotoxon). Fundus examination revealed a pink optic disk with a cup-to-disc ratio of 0.5, macular pigment regrouping, and normal blood vessels. Flash visual evoked potentials: P2 latency was normal. P2 amplitude from the left hemisphere was reduced to 50%, and P2 amplitude over the right hemisphere was normal.

**Conclusion:**

Children with genetically determined congenital anomalies need regular ophthalmic checkups to accurately assess the eye and determine the prospects of vision function development.

## 1. Introduction

Mowat-Wilson syndrome (MWS) is a genetically determined rare syndrome of congenital malformations, including craniofacial dysmorphism, developmental anomalies of the central nervous system, kidneys, heart, genitourinary system, Hirschprung disease, small head, neurodevelopmental disorders, and, although less commonly, ocular abnormalities.^[1]^ The cause of the syndrome is a mutation of the *ZEB2* gene located at 2q22.^[1–3]^ The gene encodes the transcription factor Zeb2, also called SIP1, involved in the transforming growth factor beta signaling pathway, thus being responsible for the normal course of embryogenesis.^[4]^ MWS is more common in males; the male/female ratio is approximately 1.42:1.^[5,6]^

## 2. Aim

The aim of this paper is to present the case of a 3-year-old boy with MWS and eye abnormalities.

## 3. Case report

A 3-year-old boy with MWS, confirmed by molecular genetic testing, was referred to the Division of Pediatric Ophthalmology, the K. Gibinski University Hospital Center, Medical University of Silesia, Katowice, Poland, for an ophthalmic examination.

The boy is the second child of his parents, born at 37 weeks of pregnancy with a birth weight of 2130 g and an Apgar score of 10. In the neonatal period, he had been diagnosed with polysplenia syndrome; he also had a history of grade II right and grade I left intraventricular hemorrhage.

At the age of 2 months, the child was hospitalized in the Department of Immunology due to hyperleukocytosis and agammaglobulinemia. The results of immunoglobulin tests were within the normal age-specific reference range, and the current response to vaccine antigens was found to be normal for the age. Immune responses to vaccinations have been generated. In the first year of life, the child was hospitalized in the Department of Pediatrics and Developmental Age Neurology due to facial dysmorphism and neurodevelopmental disorders. Magnetic resonance imaging of the head showed ventricular asymmetry. Electroencephalography revealed generalized abnormalities; no paroxysmal electroencephalography patterns were found. Neurological examination revealed decreased muscle tension in the head-torso axis (ax), contractures in the knee and elbow joints, and variable muscle tension with a tendency to increase. The child was also diagnosed with hypospadias, the absence of testes in the scrotum, and congenital kidney anomalies, including hypertrophied columns. Due to facial dysmorphism, neurodevelopmental disorders, and internal organ abnormalities, the child was referred for genetic consultation. A molecular examination showed 1966_1967delAT mutation in 1 allele of the *ZEB2* gene. The lesion is presented in the Human Gene Mutation Database as a pathogenic defect correlated with MWS. Gene *ZEB2*, genotype: c.1966_1967delAT/-.

A molecular examination of the coding fragment of the gene *ZEB2* (ekson fragment 8 from amino acid position: Gly306 to Tyr489 and Met656 to Lys828).

Mutation detected 1966_1967delAT (different record: p.Met656Valfs*17) in 1 allele of the gene study. The identified change is registered in the HGMD database for the *ZEB2* gene as a pathogenic mutation correlated with MWS.

Result of DNA analysis using cytogenetic microarray technique:

- oligonucleotide microarray Agilent ISCA 8 × 60K V2
- filter for the size of detected deletions and duplications—200.000 pair: arr7q34(142207461-142460503) × 3. In the tested genetic material, duplication was found in the region 7q34, size 253kbp/10 probes.

The child has regularly scheduled appointments at neurology, urology, otorhinolaryngology, genetics, hematology, and ophthalmology clinics. He also receives speech therapy, general developmental rehabilitation, and early developmental support.

**Figure 1.**
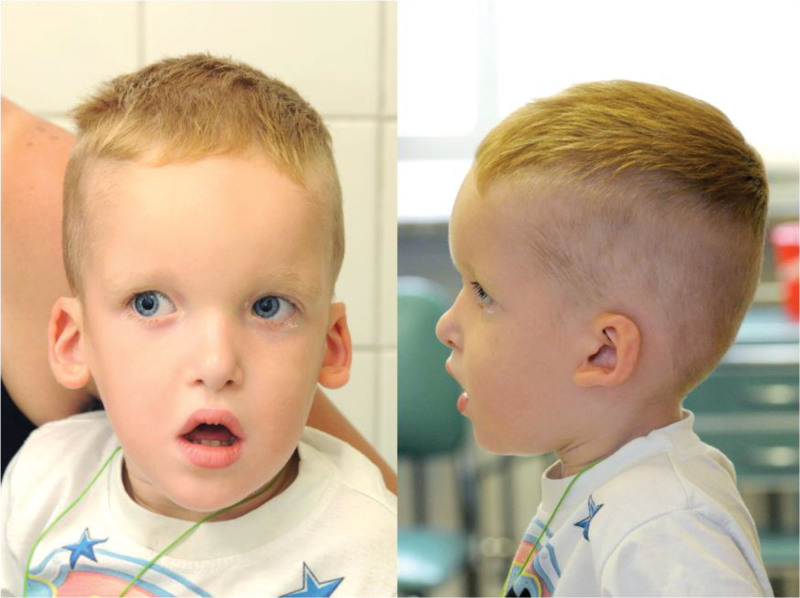
Child with Mowat-Wilson syndrome.

On the day of admission to the Division of Pediatric Ophthalmology, the child’s general condition was good. Physical examination revealed facial dysmorphism including hypertelorism, antimongoloid eye slant, deep-set eyes, a high and prominent forehead, large eyebrows, an open mouth, an M-shaped upper lip, micrognathia, and low-set, dysplastic ears (Fig. 1).

The ophthalmic examination revealed nystagmus and a normal response of pupils to light; the child followed toys with his eyes and tested well on optotypes; visual acuity was 0.05 in both eyes. The mother mentioned that the boy did not speak.

**Figure 2.**
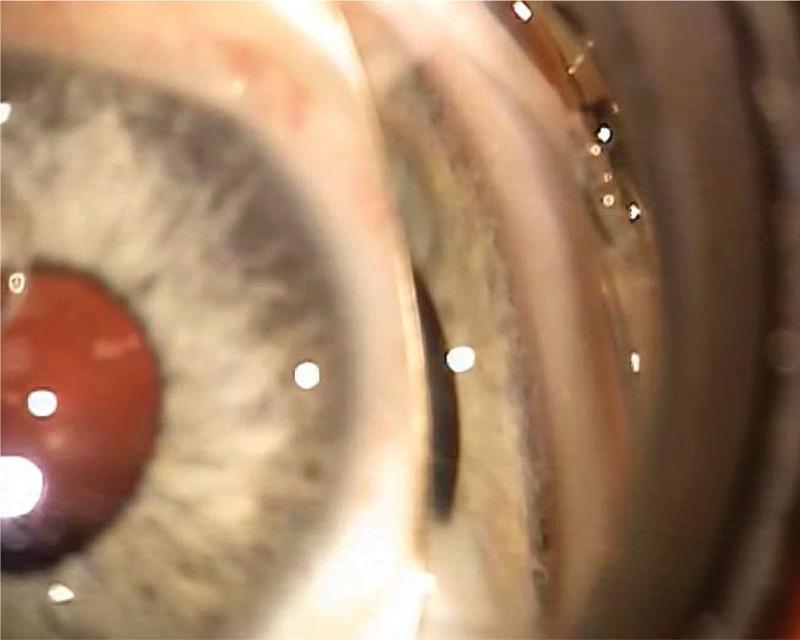
Gonioscopy of the eye.

**Figure 3.**
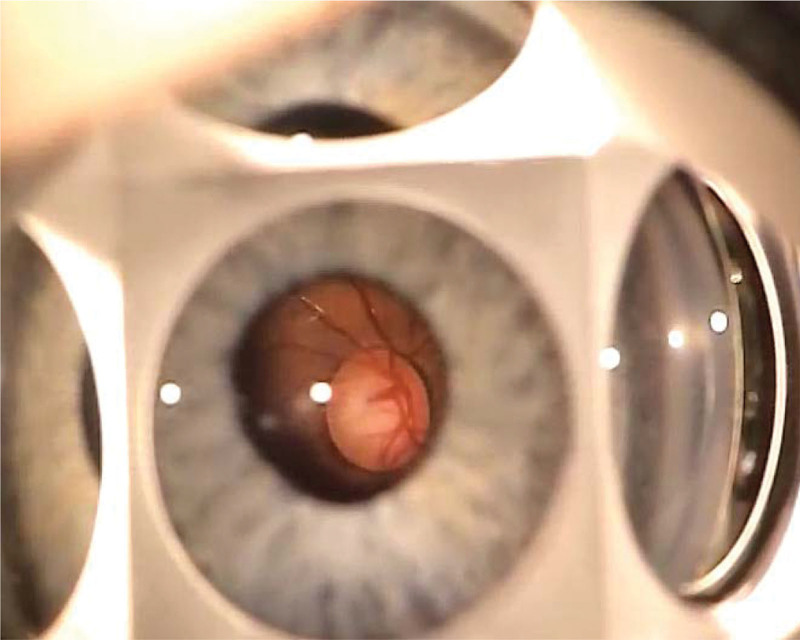
Fundus of the eye.

The examination was continued under general anesthesia. Eye optical systems were transparent, corneal diameters were 10.5 × 11.5 mm, and intraocular pressure was normal, that is, 17 and 18 mm Hg in the right and left eye, respectively. Corneal thickness was 606 µm in the right eye and 588 µm in the left eye. Gonioscopy showed a prominent and anteriorly displaced line of Schwalbe in the temporal quadrants (posterior embryotoxon). Fundus examination revealed a pink optic disk with a cup-to-disc ratio of 0.5, macular pigment regrouping, and normal blood vessels (Figs. 2 and 3).

**Figure 4.**
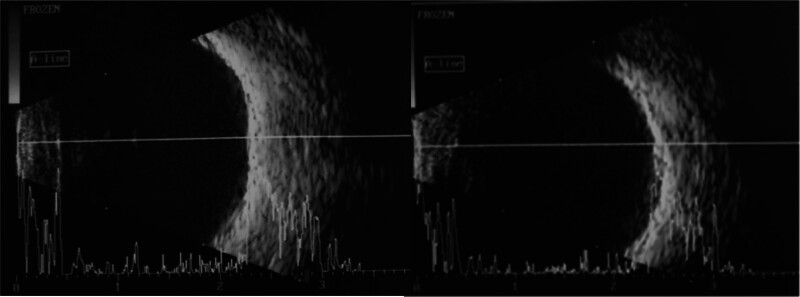
Ocular ultrasonography.

Ocular ultrasonography showed normal posterior segments (Fig. 4) and ax lengths of 23 and 22.87 mm in the right and left eye, respectively. Refraction of the optical system after accommodation paralysis was + 0.75 spherical diopter + 0.25 cylinder diopter ax 78° in the right and + 0.75 spherical diopter + 0.25 cylinder diopter ax 73° in the left eye. Flash visual evoked potentials P2 latency was normal. P2 amplitude from the left hemisphere was reduced to 50%, and P2 amplitude over the right hemisphere was normal. Alternating divergent strabismus was also found.

Following diagnostic testing, the child was discharged home in good general condition. Due to nystagmus, strabismus, and changes in the appearance of the optic disc, further diagnostic procedures were scheduled in the Outpatient Department. Regular checkups with other medical specialists were also recommended.

## 4. Discussion

MWS is a rare genetic congenital malformation syndrome. According to the National Organization for Rare Disorders, the prevalence of MWS is 1 per 50,000 to 100,000 live births. Coyle et al^[7]^ analyzed 256 cases of MWS reported worldwide by 2015. However, the prevalence may be higher due to undiagnosed cases, including patients who are not fully symptomatic.

When describing the ocular lesions characteristic of MWS, Mowat and Wilson et al^[1,8]^ emphasized hypertelorism and deeply-set but large eyes. The eyebrows tend to be broad and horizontal, with a wide medial separation. The authors also mention early nystagmus owing to fixation difficulties. Some patients with blue irides were shown to have dark pigment clumps in the irides, described as heterochromia. Garavelli and Mainardi^[6]^ reported that 4.1% of patients with MWS had structural anomalies of the eye. Adam et al^[9]^ mentioned that 4% of affected individuals had eye anomalies, including microphthalmia, iris and retinal colobomas, Axenfeld anomaly, peripupillary atrophy, ptosis, cataract, retinal aplasia, nystagmus, and strabismus. Mowat and Wilson^[1]^ described 1 case of unilateral eyelid ptosis. Vascular and retinal fissures were also found. Faltin et al^[3]^ described a 3-month-old girl with unilateral coloboma of the iris and retina and other symptoms typical of MWS. Ariss et al^[10]^ reported a 9-year-old girl with MWS and severe ocular lesions, including bilateral microphthalmia, cataract, and retinal aplasia. Other authors also pointed out the occurrence of changes in the organ of vision. Hartill et al^[11]^ described a case of a 41-week-old child with deep-set eyeballs, diagonal wrinkles, and strabismus, while Tanteles et al^[12]^ described 2 patients with vision defects. In the first patient with poor visual contact, the ophthalmic examination revealed bilateral colobomata of the iris and choroid and optic nerve involvement. The other patient had bilateral microphthalmia, right iris coloboma, and left partial aniridia.

The presented patient remains under the supervision of several health professionals due to multi-organ structural anomalies and functional disorders in the form of psychomotor and speech delays. The clinical picture prompted the attending physicians to order a genetic test, which revealed *ZEB2* gene mutation indicative of MWS. Ocular anomalies potentially associated with MWS are quite diverse. Our patient exhibited abnormalities reported by the majority of authors, that is, hypertelorism, deep-set eyeballs, nystagmus, and strabismus. Gonioscopy revealed displacement of the Schwalbe line anterior to the limbus of the cornea (posterior embryotoxon), which is characteristic of the Axenfeld anomaly observed in patients with MWS.^[9]^ Due to ocular disorders, the boy is under regular supervision by our ophthalmic clinic.

## 5. Conclusion

Although only about 4% of patients diagnosed with MWS exhibit ocular abnormalities, they should receive regular ophthalmic evaluations to accurately assess the organ of vision and determine the prospects of vision function development.

## Author contributions

**Conceptualization:** Bogumiła Wójcik-Niklewska.

**Formal analysis:** Bogumiła Wójcik-Niklewska, Erita Filipek.

**Investigation:** Bogumiła Wójcik-Niklewska.

**Methodology:** Bogumiła Wójcik-Niklewska.

**Software:** Bogumiła Wójcik-Niklewska.

**Visualization:** Bogumiła Wójcik-Niklewska.

**Writing – original draft:** Bogumiła Wójcik-Niklewska.

**Writing – review & editing:** Bogumiła Wójcik-Niklewska.

**Supervision:** Erita Filipek.
